# Predictors and outcomes of obstructive sleep apnea in patients with chronic obstructive pulmonary disease in China

**DOI:** 10.1186/s12890-021-01780-4

**Published:** 2022-01-04

**Authors:** Pan Zhang, Bi Chen, Heqing Lou, Yanan Zhu, Peipei Chen, Zongmei Dong, Xuan Zhu, Ting Li, Peian Lou

**Affiliations:** 1 Department of Control and Prevention of Chronic Non-communicable Diseases of Xuzhou Center for Disease Control and Prevention, 142 West Erhuan Road, Xuzhou, Jiangsu China; 2 Department of Respiratory Medicine, Affiliated Hospital of Xuzhou Medical University, Xuzhou, China

**Keywords:** Chronic obstructive pulmonary disease, Obstructive sleep apnea, Risk factor, Scale, Polysomnography

## Abstract

**Background:**

“Overlap syndrome” refers to obstructive sleep apnea (OSA) combined with chronic obstructive pulmonary disease (COPD), and has poorer outcomes than either condition alone. We aimed to evaluate the prevalence and possible predictors of overlap syndrome and its association with clinical outcomes in patients with COPD.

**Methods:**

We assessed the modified Medical Research Council dyspnea scale (mMRC), Epworth sleepiness scale (ESS), COPD assessment test (CAT), Hospital Anxiety and Depression Scale (HADS), Charlson Comorbidity Index (CCI), and STOP-Bang questionnaire (SBQ) and performed spirometry and full overnight polysomnography in all patients. An apnea–hypopnea index (AHI) ≥ 5 events per hour was considered to indicate OSA. Risk factors for OSA in COPD patients were identified by univariate and multivariate logistic regression analyses.

**Results:**

A total of 556 patients (66%) had an AHI ≥ 5 events per hour. There were no significant differences in age, sex ratio, mMRC score, smoking index, number of acute exacerbations and hospitalizations in the last year, and prevalence of cor pulmonale between the two groups (all *p* > 0.05). Body mass index (BMI), neck circumference, CAT score, CCI, ESS, HADS, and SBQ scores, forced expiratory volume (FEV)_1_, FEV_1_% pred, FEV_1_/forced vital capacity ratio, and prevalence of hypertension, coronary heart disease, and diabetes were all significantly higher and the prevalence of severe COPD was significantly lower in the COPD-OSA group compared with the COPD group (*p* < 0.05). BMI, neck circumference, ESS, CAT, CCI, HADS, hypertension, and diabetes were independent risk factors for OSA in COPD patients (*p* < 0.05). SBQ could be used for OSA screening in patients with COPD. Patients with severe COPD had a lower risk of OSA compared with patients with mild or moderate COPD (β =  − 0.459, odds ratio = 0.632, 95% confidence interval 0.401–0.997, *p* = 0.048).

**Conclusion:**

Patients with overlap syndrome had a poorer quality of life, more daytime sleepiness, and a higher prevalence of hypertension and diabetes than patients with COPD alone. BMI, neck circumference, ESS, CAT, CCI, HADS, hypertension, and diabetes were independent risk factors for OSA in patients with COPD. The risk of OSA was lower in patients with severe, compared with mild or moderate COPD.

## Background

Chronic obstructive pulmonary disease (COPD) is a common, preventable and treatable disease characterized by chronic airflow limitation, which is not fully reversible [1]. Approximately 299.4 million people have suffered from COPD, which was responsible for about 3.2 million deaths worldwide, and COPD was the fifth leading cause of loss of disability-adjusted life years (DALYs) in 2017 [2]. COPD affected around 13.6%–13.7% of adults aged 40 or older in China according to two recent investigations [3, 4], with a mortality rate of 68 per 100,000 people in 2017, representing the fourth leading cause of DALYs in China [5].

Obstructive sleep apnea (OSA) is another common respiratory disease, which is characterized by recurrent closure of the upper airway during sleep and is usually associated with COPD. The coexistence of OSA and COPD has been described as “overlap syndrome” [6]. The prevalence of overlap syndrome varies among geographic regions and populations [7]. Shawon et al. [7] reported incidences of COPD coexisting with OSA ranging from 2.9% to 65.9% in a systematic review. Overlap syndrome has more serious adverse effects on the quality of life in patients with COPD [7, 8]. Because of their similar pathophysiological effects, especially in terms of hypoxia and systemic inflammation, the simultaneous occurrence of COPD and OSA has more severe nocturnal hypoxemic and hypercarbia effects than either COPD or OSA alone, and is more likely to be complicated with cardiovascular diseases [7, 9]. Overlap of these two conditions can also reduce daytime oxygen saturation and quality of life-related scores, and increase the frequencies of acute exacerbation, comorbidity, economic burden and mortality due to COPD [7, 10, 11]. However, continuous positive airway pressure treatment can reduce the risk of COPD exacerbation and prolong patient survival [7, 11, 12]. The identification and timely treatment of overlap syndrome in patients with COPD therefore can help to improve patient prognosis. Many studies have investigated the possible predictive factors of overlap syndrome; however, the findings have been inconsistent. Peripheral edema and emphysema have been reported to predict OSA [13, 14], but the predictive values of age, sex, body mass index (BMI), neck circumference, and smoking status remained controversial [15, 16]. Further investigations are, therefore, needed to clarify the roles of these factors in predicting OSA. Furthermore, a better understanding of the important risk factors and clinical outcomes of patients with overlap syndrome in different geographical areas would help the relevant health authorities to implement targeted intervention strategies [17]. We conducted a cross-sectional community-based study in China to clarify the prevalence, clinical characteristics, risk factors of COPD with OSA, and the relationship between airflow limitation in COPD and severity of OSA.

## Methods

### Study design

This was a cross-sectional study conducted in 11 regions of the Xuzhou area in eastern China from December 2018 to December 2019. The aim was to investigate the prevalence of OSA in patients with COPD and to identify its predictors. Sampling was carried out by multistage cluster random sampling from all 11 regions in the study area. In the first stage, three community health service centers were selected from each region by cluster random sampling; in the second stage, three community health service stations were selected by cluster random sampling from each selected community health service center; and in the final stage, patients with COPD who met the inclusion criteria and who were registered at the health service stations were enrolled, according to their medical records. Information on all the variables was collected by trained investigators by face-to-face interview on the day of the participants’ regular medical appointments at the health centers.

### Participants

**Fig. 1:**
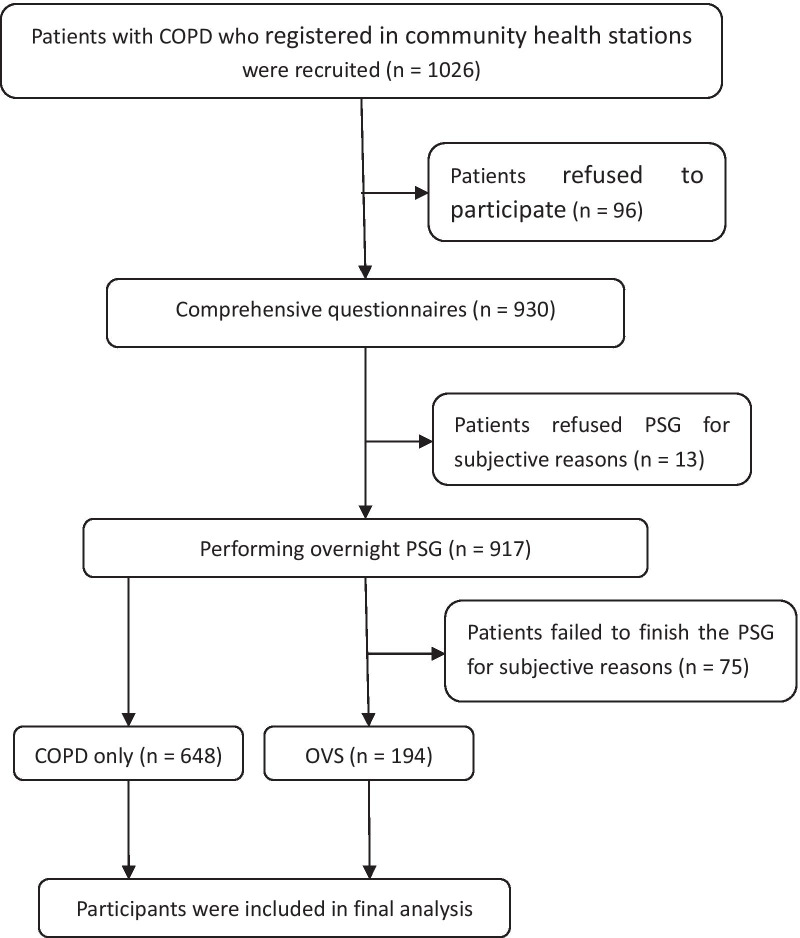
Flowchart of the study design

Patients diagnosed with COPD with severity classified according to the 2017 Global Initiative for Chronic Obstructive Lung Disease (GOLD) guidelines were eligible [1]. Exclusion criteria were patients who refused to participate in the study, patients who failed to perform pulmonary function test due to acute exacerbation or severe illness, patients unable to tolerate or who refused whole-night polysomnography, patients disqualified from polysomnography, and pregnant women (Fig. 1).

The study protocol was approved by the medical ethics committee of Xuzhou Center for Disease Control and Prevention (number: 2012010). All patients signed written informed consent, underwent pulmonary function tests and polysomnography, and provided the required information. The trial was conducted according to the 2000 revised version of the Helsinki Declaration. The study followed the STROBE (Strengthening the Reporting of Observational Studies in Epidemiology) guidelines for observational studies [18].

### Spirometry

Spirometry was performed at health centers using a portable spirometer (Medikro PRO, Finland). The pulmonary function testing was performed in a comfortable and quiet environment. Before the test, the subjects were explained the purpose of the test and the essentials of the action, and asked whether they met the exclusion criteria of pulmonary function test. The pulmonary instrument was adjusted and calibrated the parameters before each measurement. The basic pulmonary function and lung function after bronchodilator test were measured by a unified method. After completing the basic test, every person used salbutamol aerosol 400ug. A 15-min interval after the bronchodilator test, all subjects were measured the pulmonary function. With the automated device, 3 readings were taken at 1 min intervals. The difference between FEV1 and FVC were less than 200 ml, and PEF was less than 0.67L/s. COPD was defined according to GOLD guidelines according to the ratio of post bronchodilator forced expiratory volume (FEV) in 1 s (FEV_1_) to forced vital capacity (FVC) < 70% [1].

### Monitoring with polysomnography and OSA severity

Participants who met the diagnostic criteria for COPD were monitored at home for at least 7 h at night using a comprehensive portable polysomnography machine (PSM-100A, Sealand, Chengdu, China). The data including snoring, nasal airflow, thoracoabdominal breathing exercise, blood oxygen saturation and disorder index by the monitor were automatically recorded and analyzed, and then manually corrected the next day. The results were recorded by two skilled sleep technicians, following standard criteria [19]. OSA was defined as an apnea–hypopnea index (AHI) ≥ 5 events per hour. OSA’s severity was categorized based on the AHI as normal (< 5), mild (5–14.9), moderate (15–29.9) and severe (≥ 30) [20].

### COPD clinical outcomes and other variables

The clinical outcomes of patients with COPD included dyspnea and health status. Dyspnea was measured using the modified Medical Research Council mMRC dyspnea scale [21] with a score of 0–4 points, with a higher score indicating more-severe dyspnea. Health status was measured using the COPD assessment test (CAT) [22], which included a total of eight items, each with a score of 0–5, giving a total score of 0–40, with a higher score indicating a poorer health status. OSA was measured using the STOP-Bang questionnaire (SBQ), including eight questions with ‘yes’ (1 point) or ‘no’ (0 points) answers, with a total score ≥ 3 indicating OSA [23].

Depression and anxiety were screened and measured using the 14-item Hospital Anxiety and Depression Scale (HADS), including seven items relating to anxiety (HADS-A) and the other seven relating to depression (HADS-D). The overall score ranged from 0 to 21, with a score ≥ 8 on a subscale indicating possible HADS-A or HADS-D, respectively [24]. Comorbidities were assessed according to the Charlson Comorbidity Index (CCI) [25], which included 17 common concomitant medical conditions, as follows: (1) 1 point: myocardial infarction, heart failure, peripheral vascular disease, cerebrovascular disease, dementia, chronic lung disease, connective tissue disease, peptic ulcer, mild liver disease and diabetes without organ damage; (2) 2 points: hemiplegia, moderate to severe kidney disease, diabetes mellitus with organ injury, solid tumor in any part, malignant lymphoma and leukemia; (3) 3 points: moderate and severe liver disease; and (4) 6 points: metastatic solid tumor, acquired immune deficiency syndrome. Age was also scored as 1 point for 50–59 years old, 2 points for 60–69 years old, and 3 points for ≥ 70 years old. The CCI score plus age score was taken as the total score. In this study, cases of hypertension, coronary heart disease, pulmonary heart disease, and diabetes were separately counted again.

### Other demographic variables

Independent variables included patients with COPD and OSA/COPD overlap syndrome. Subject-specific variables collected using a self-designed questionnaire included sex, age, BMI, neck circumference, course of disease, smoking index, number of acute exacerbation in the past year, and number of acute exacerbations requiring hospitalization. BMI was calculated by dividing body weight in kilograms by height in meters squared. Smoking index was equal to the number of cigarettes per day multiplied by the number of years of smoking.

### Sample-size calculation

Assuming an estimated 40.0% of participants with OSA/COPD overlap syndrome [9] with an admissible error of 10%, power of 90%, and alpha of 0.05, and allowing for a refusal rate of 15%, we calculated that a minimum of 663 subjects needed to be enrolled. The COPD registry system covered all communities in the Xuzhou area. A total of 29,800 patients with COPD were registered in 2950 health service stations from 211 community health service centers, with about 10 patients with COPD in each community health service station.

### Primary and secondary outcomes

The primary outcomes were influence factors for overlap syndrome. The secondary outcome was the prevalence of overlap syndrome in patients with COPD.

### Statistical analysis

Risk factors for COPD with coexisting OSA were analyzed using SPSS 17.0(SPSS Inc., Chicago, USA). Participants were divided into two groups according to the results of monitoring with polysomnography. The COPD group comprised patients with an AHI < 15 events per hour, and the COPD-OSA group included patients with an AHI ≥ 15 events per hour. Categorical data were expressed as number of cases (percentage) and analyzed using χ^2^ tests. Measured data were tested for normality, and data conforming to a normal distribution were expressed as mean ± standard deviation, with *t*-tests for pairwise comparisons. Data that did not conform to a normal distribution were expressed as median (interquartile range), and differences were compared by Mann–Whitney U tests. Correlations between two continuous variables were analyzed by Spearman’s correlation analysis.

Multivariate logistic regression analysis (dependent variables: 0, patients with COPD without risk of OSA symptoms (AHI < 15 events per hour); 1, patients with COPD with risk of OSA symptoms (AHI ≥ 15 events per hour)) were performed to assess the likelihood of risk of OSA versus no risk of OSA in patients with COPD, in the presence of exposure variables including age, sex, BMI, neck circumference, smoking index, FEV_1_, FEV_1_% of expected value, FEV1 (%predicted) < 50%, FEV_1_/FVC, CAT score, Epworth sleepiness scale (ESS) score, SBQ score, HADS-A score, HADS-D score, hypertension, coronary heart disease, and diabetes. FEV_1_, FEV_1_% of expected value, FEV_1_/FVC, SBQ, HADS-A, and HADS-D were assigned a value of 1 if they were greater than the average and 0 if they were less than the average. FEV_1_% accounting for a predicted value < 50% was also assigned a score of 1 and FEV_1_% accounting for a predicted value ≥ 50% was assigned a score of 0. All tests were two sided. A *p*-value < 0.05 was considered statistically significant.

## Results

### Characteristics of study participants and OSA symptoms in the participants

A flowchart of the study design and process is shown in Fig. 1. A total of 1026 patients with COPD registered in 99 community health stations were selected for this observational study. Based on the design principles, 184 subjects were excluded, including 96 who refused to participate, 47 without pulmonary function tests because of acute exacerbation or serious condition, 28 who were unable to tolerate whole-night polysomnography, and 13 who refused to be monitored by polysomnography. A total of 842 patients were therefore included in the final analysis (606 males (72%) and 236 females (28%), average age 63 ± 8 years). A total of 66.0% of the 842 patients completed the study with an AHI ≥ 5 events per hour.

### Characteristics of participants with and without risk of OSA symptoms

The general characteristics of the patients according to AHI are shown in Table 1. There were no significant differences in age, sex, smoking index, number of acute exacerbations in the year before admission, hospital frequency, mMRC score, and cor pulmonale between the COPD group and COPD-OSA group (all *p* > 0.05). BMI, neck circumference, FEV1, FEV1 (%predicted), FEV1/FVC, AHI, CAT, ESS, SBQ, HADS-A, and HADS-D scores, and the incidences of hypertension, coronary heart disease, and diabetes were all significantly higher and the proportion of severe COPD [(FEV1 (%predicted) < 50%] was significantly lower in the COPD-OSA group compared with the COPD group (all *p* < 0.05).

**Table 1:** Comparison of general characteristics of patients between two groups (Mean ± SD)

Variables	COPD group (N = 648)			COPD overlap OSA group(N = 194)			χ^2^/t value	P
	AHI < 5 events / h	5 events / h ≤ AHI < 15 events / h	Total	15 events / h ≤ AHI < 30 events / h	AHI ≥ 30 events / h	Total		
Gender (male,%)	200(69.93%)	253(69.89%)	453(69.91%)	87(70.73%)	54(76.06%)	141(72.68%)	0.427	0.513
Age y (mean ± SD)	62 ± 7	64 ± 9	63 ± 8	63 ± 9	64 ± 9	62 ± 7	1.57	0.117
BMI (kg/m^2^) (mean ± SD)	21 ± 3	22 ± 4	22 ± 4	23 ± 4	24 ± 4	24 ± 4	− 6.11	< 0.001
Neck circumference (cm) (mean ± SD)	36.5 ± 2.2	37.3 ± 3.1	37.0 ± 2.6	37.5 ± 35	38.6 ± 3.6	38.1 ± 3.5	3.97	< 0.001
Smoking index (package year)	32.5 ± 27.8	37.5 ± 27.0	35.5 ± 27.5	38.5 ± 25.5	40.5 ± 22.6	39.2 ± 24.8	− 1.68	0.093
Smoke status(never,%)	123(43.01%)	133(36.74%)	248(38.27%)	42(34.15%)	25(35.21%)	67(34.54%)	5.53	0.06
FEV_1_ (L) (mean ± SD)	\	1.1 ± 0.8	1.0 ± 0.8	1.2 ± 1.0	1.4 ± 1.3	1.3 ± 1.1	− 4.18	< 0.001
FEV_1_% of expected value (mean ± SD)	42.0 ± 28.5	45.5 ± 30.0	44.5 ± 29.5	52.5 ± 32.6	60.0 ± 39.5	55.0 ± 36.5	− 4.11	< 0.001
FEV_1_% of predicted value < 50% ( n)	169(59.09%)	190(52.49%)	359(55.40%)	51(41.46%)	24(33.80%)	75(38.66%)	10.047	0.002
FEV_1_/FVC(%)	38.0 ± 21.0	40.0 ± 20.0	39.0 ± 21.0	43.0 ± 23.0	55.0 ± 25.0	49.0 ± 240	− 5.624	< 0.001
AHI (events / h)	2.5 ± 2.2	9.1 ± 5.2	6.2 ± 5.7	19.8 ± 7.3	37.8 ± 20.0	26.7 ± 14.7	− 28.987	< 0.001
Acute exacerbation in the past year (frequency)	2.1 ± 1.5	2.2 ± 2.1	2.1 ± 2.0	2.1 ± 2.0	2.1 ± 2.0	2.0 ± 2.1	0.60	0.546
Hospitalization in the past year (frequency)	0.5 ± 1.1	1.3 ± 2.2	1.3 ± 1.5	1.2 ± 2.1	1.1 ± 2.0	1.2 ± 2.0	0.75	0.453
mMRC scores (mean ± SD)	2.0 ± 0.4	1.0 ± 2.2	1.5 ± 1.3	1.8 ± 2.0	1.5 ± 1.3	1.6 ± 1.1	− 0.97	0.331
CAT scores (mean ± SD)	14.7 ± 6.5	16.8 ± 7.3	15.9 ± 7.2	17.3 ± 8.2	19.2 ± 8.4	18.4 ± 8.4	− 4.08	< 0.001
ESS scores (mean ± SD)	4.1 ± 4.0	5.2 ± 4.1	5.1 ± 4.0	6.2 ± 5.1	8.1 ± 9.0	7.8 ± 5.9	− 7.32	< 0.001
SBQ scores(mean ± SD)	3.1 ± 2.0	4.4 ± 3.2	4.2 ± 3.0	5.5 ± 4.0	7.0 ± 4.0	6.0 ± 4.0	− 7.50	< 0.001
HADS-A scores	4.5 ± 3.2	6.5 ± 4.1	5.7 ± 3.8	7.6 ± 4.0	10.5 ± 7.5	9 .3 ± 6.1	− 9.92	< 0.001
HADS-D scores	4.4 ± 2.7	6.3 ± 4.3	5.5 ± 3.6	7.2 ± 4.5	11.0 ± 7.2	9.5 ± 6.4	− 11.10	< 0.001
CCI scores (mean ± SD)	2.0 ± 1.0	3.0 ± 2.0	2.7 ± 1.4	3.0 ± 1.5	4.0 ± 2.0	3.4 ± 1.7	0	1
Hypertension (n,%)	71(24.83%)	119(32.87%)	190(29.32%)	52(42.28%)	41(57.75%)	93(47.94%)	8.001	0.005
Cor Pulmonale (n,%)	15(5.24%)	22(6.08%)	37(5.71%)	9(7.32%)	6(8.45%)	15(7.73%)	0.937	0.333
Coronary heart disease(n,%)	22(7.69%)	45(12.43%)	67(10.34%)	14(11.38%)	6(8.45%)	20(10.31%)	7.305	0.007
Diabetes (n,%)	15(5.24%)	61(16.85%)	76(11.73%)	24(19.51%)	20(28.17%)	44(22.68%)	13.845	< 0.001

Gender, FEV1% of predicted value < 50%, Hypertension, cor pulmonale,coronary heart disease and diabetes are presented as n; other values are the mean with SD; *COPD* chronic obstructive pulmonary disease; *OSA* obstructive sleep apnea; *SD* standard deviation; *BMI* body mass index; *FEV*_1_ forced expiratory volume in 1 s; *FVC* forced vital capacity; *AHI* apnea–hypopnea index; *mMRC* Modified British Medical Research Council; *CAT* COPD assessment test; *ESS* Epworth sleepiness scale; *CCI* Charlson comorbidity index; *SBQ* STOP-Bang; *HADS-A* Hospital Anxiety and Depression Scale for anxiety; *HADS-D* Hospital Anxiety and Depression Scale for depression

### Correlations of AHI and risk factors

Analysis of the correlations between AHI and variables revealed significant differences between the groups in terms of BMI, neck circumference, FEV_1_, percentage of FEV_1_ accounting for predicted value, percentage of FEV_1_ accounting for predicted value < 50%, CAT, ESS, SBQ, HADS-A, and HADS-D scores, hypertension, coronary heart disease, and diabetes (all *p* < 0.05). There was a significant negative correlation between AHI and FEV_1_% accounting for predicted value < 50% (*p* < 0.05) (Table 2).

**Table 2:** Correlation analysis of AHI and risk factors

Variables	BMI	Neck circumference	FEV_1_	FEV_1_% of expected value	FEV_1_ (%predicted) < 50%	CAT scores	ESS scores	SBQ scores	HADS-A scores	HADS-D scores	Hypertension	Coronary heart disease	Diabetes
r values	0.281	0.167	0.177	0.188	− 0.165	0.242	0.269	0.271	0.456	0.447	0.221	0.154	0.385
p values	< 0.001	0.001	< 0.001	< 0.001	0.001	< 0.001	< 0.001	< 0.001	< 0.001	< 0.001	0.001	0.035	< 0.001

*BMI* body mass index; *FEV*_1_ forced expiratory volume in 1 s; *CAT* COPD assessment test; *ESS* Epworth sleepiness scale; *SBQ* STOP-Bang; *HADS-A* Hospital Anxiety and Depression Scale for anxiety; *HADS-D* Hospital Anxiety and Depression Scale for depression

### Independent predictors for risk of OSA symptoms in COPD

The risk factors for coexisting OSA in patients with COPD were analyzed by univariate logistic regression analysis. COPD coexisting with OSA (AHI ≥ 15 events per hour) was taken as the dependent variable and assigned a score of 1 and AHI < 15 events per hour was assigned a score of 0. Age, BMI, neck circumference, FEV_1_, FEV_1_% of expected value, FEV_1_ (%predicted) < 50%, CAT, ESS, SBQ, HADS-A, HADS-D, hypertension, coronary heart disease, and diabetes were taken as independent variables and analyzed one by one by univariate logistic regression. BMI, neck circumference, FEV_1_, CAT, ESS, SBQ, HADS-A, HADS-D, hypertension, coronary heart disease, and diabetes were all shown to be risk factors for COPD combined with OSA (all *p* < 0.05) (Table 3). The risk of OSA in patients with severe COPD [FEV_1_ (%predicted) < 50%] was lower than that in patients with mild and moderate COPD[FEV_1_ (%predicted) ≥ 50%] (β =  − 0.621, odds ratio (OR) = 0.551, 95% confidence interval (CI): 0.412–0.691, *p* < 0.001) (Table 3). Patients with COPD and coexisting OSA had poorer ESS, SBQ, HADS-A, HADS-D, and CAT scores, and more hypertension, coronary heart disease, and diabetes.

**Table 3:** Results of univariate logistic regression used to explore risk factors for COPD coexisting OSA (AHI > 15 times per hour)

Variables	β	*Wald*	*P*	OR	95% CI
Age	0.019	2.017	0.142	1.017	0.992 ~ 1.041
BMI (kg/m^2^)	0.178	32.158	< 0.001	1.186	1.115 ~ 1.258
Neck circumference	0.186	12.452	< 0.001	1.201	1.114 ~ 1.289
FEV_1_	0.006	2.457	0.047	1.009	1.000 ~ 1.019
FEV_1_% of expected value	0.025	0.141	0.463	0.958	0.841 ~ 1.075
FEV_1_ (%predicted) < 50%	− 0.621	7.412	0.041	0.551	0.412 ~ 0.691
CAT scores	0.201	32.263	< 0.001	1.305	1.121 ~ 1.490
ESS scores	0.137	9.524	< 0.001	1.128	1.074 ~ 1.182
SBQ scores	0.223	17.623	< 0.001	1.311	1.125 ~ 1.498
HADS-A scores	0.243	29.132	< 0.001	1.324	1.131 ~ 1.517
HADS-D scores	0.251	30.541	< 0.001	1.301	1.122 ~ 1.481
Hypertension	0.351	35.022	0.029	2.011	1.551 ~ 2.470
Coronary heart disease	0.133	5.851	0.045	1.148	1.006 ~ 1.291
Diabetes	0.402	37.112	< 0.001	2.055	1.442 ~ 2.669

Overlap syndrome (AHI > 15 times per hour) was considered as the dependent variable and age, BMI, neck circumference, FEV_1_, FEV_1_% of expected value, FEV_1_(%predicted) < 50%, CAT score, ESS score, SBQ scores, HADS-A scores, HADS-D scores, hypertension, coronary heart disease and diabetes as independent variables. *BMI* body mass index; *FEV*_1_ forced expiratory volume in 1 s; *CAT* COPD assessment test; *ESS* Epworth sleepiness scale; *SBQ* STOP-Bang; *HADS-A* Hospital Anxiety and Depression Scale for anxiety; *HADS-D* Hospital Anxiety and Depression Scale for depression

We also analyzed the risk factors for COPD coexisting with OSA by multivariate logistic regression analysis. BMI, neck circumference, CAT, ESS, HADS-A, HADS-D, and CCI scores, and FEV_1_ (%predicted) < 50% o were used as independent variables in multivariate logistic regression using the forced entry method, after adjusting for age, sex, smoking index and FEV_1_. BMI (β = 0.153, OR = 1.165, 95% CI: 1.094–1.242, *p* < 0.001), neck circumference, CAT, ESS, SBQ, HADS-A, HADS-D, and CCI were all identified as independent risk factors for OSA in patients with COPD. Hypertension and diabetes were also identified as independent risk factors of OSA in patients with COPD after adjusting for age, sex, smoking index FEV_1_, BMI, CAT, ESS, HADS-A, HADS-D, and percentage of FEV_1_ accounting for < 50% of the predicted values. SBQ was shown to be an independent risk factor after adjusting for age, smoking index, FEV_1_, CAT, ESS, HADS-A, HADS-D, CCI, and FEV_1_ (%predicted) < 50%. The risk of OSA in patients with severe COPD [FEV_1_ (%predicted) < 50%] was lower than that in patients with mild and moderate COPD (β =  − 0.549, OR = 0.578, 95% CI: 0.392–0.782, *p* = 0.045) (Table 4).

**Table 4:** Multivariate logistic regression analysis of risk factors for COPD coexisting OSA (AHI > 15 times per hour)

Variables	β	*Wald*	*P*	OR	95% CI
BMI (kg/m^2^)	0.161	26.794	< 0.001	1.175	1.104 ~ 1.251
Neck circumference	0.101	4.251	< 0.001	1.099	1.013 ~ 1.211
FEV_1_	0.023	2.086	0.149	1.023	0.991 ~ 1.057
FEV_1_ (%predicted) < 50%	− 0.549	5.893	0.045	0.578	0.392 ~ 0.782
CAT scores	0.187	30.154	< 0.001	1.195	1.112 ~ 1.288
ESS scores	0.118	7.667	< 0.001	1.113	1.052 ~ 1.177
SBQ scores	0.188	13.866	< 0.001	1.206	1.119 ~ 1.301
HADS-A	0.201	27.956	< 0.001	1.275	1.126 ~ 1.432
HADS-D	0.198	28.012	< 0.001	1.243	1.120 ~ 1.401
Hypertension	0.309	30.442	0.037	1.831	1.357 ~ 2.321
Coronary heart disease	0.111	3.458	0.053	1.117	0.999 ~ 1.249
Diabetes	0.311	34.521	< 0.001	1.852	1.421 ~ 2.312

Overlap syndrome (AHI > 15 times per hour) was considered as the dependent variable and age, BMI, neck circumference, FEV_1_, FEV_1_(%predicted) < 50%, CAT score, ESS score, SBQ scores, HADS-A scores, HADS-D scores, hypertension, coronary heart disease and diabetes as independent variables. *BMI* body mass index; *FEV*_1_ forced expiratory volume in 1 s; *CAT* COPD assessment test; *ESS* Epworth sleepiness scale; *SBQ* STOP-Bang; *HADS-A* Hospital Anxiety and Depression Scale for anxiety; *HADS-D* Hospital Anxiety and Depression Scale for depression.

### Correlation between airflow limitation of COPD and severity of OSA

We analyzed the relationship between the degree of airflow restriction according to GOLD grading and the severity of OSA according to AHI. There were significant differences in the severity of airflow restriction in patients with COPD according to the distribution of AHI (< 5, ≥ 5 to < 15, ≥ 15 to < 30, and ≥ 30 events per hour) (Table 5). The proportion of AHI < 5 events per hour increased with increasing airflow restriction (*p* < 0.001), while the proportions of 15 to < 30 and ≥ 30 events per hour decreased with increasing airflow restriction (both *p* < 0.001). Compared with COPD patients with mild and moderate airflow restriction [FEV_1_(%predicted) ≥ 50%], the proportion of AHI < 5 events per hour was higher in COPD patients with severe airflow restriction [FEV_1_(%predicted) < 50%] (*p* < 0.001), while the proportion of AHI ≥ 30 events per hour was lower in COPD patients with severe airflow restriction [FEV_1_(%predicted) < 50%] (*p* < 0.001).

**Table 5:** Correlation between the degree of airflow limitation in COPD (GOLD classification) and the severity of OSA (n, %)

GOLD classification	Cases (n = 842)	AHI < 5 times / h (n = 286)	5 times / h ≤ AHI < 15 times / h (n = 362)	15 times / h ≤ AHI < 30 times / h (n = 123)	AHI ≥ 30 times / h (n = 71)
I	110(13.06%)	19(6.64%)	49(13.54%)	22(17.89%)	20(28.17%)
II	320(38.00%)	94(32.87%)	148(40.88%)	47(38.21%)	31(43.66%)
III	304(36.10%)	124(43.36%)	127(35.08%)	38(30.89%)	15(21.13%)
IV	108(12.83%)	49(17.13%)	38(10.50%)	16(13.01%)	5(7.04%)
χ^2^ value	–	29.247	4.363	3.652	21.035
P value	–	< 0.001	0.225	0.302	< 0.001

*AHI* apnea–hypopnea index;

## Discussion

In this cross-sectional study, we assessed the prevalence, possible predictors, and associated comorbidities of overlap syndrome in patients with COPD.

The prevalence of COPD coexisting with OSA was 66.0%, based on a threshold AHI ≥ 5 events per hour, while 23% had an AHI ≥ 15 events per hour. Compared with COPD patients without OSA, patients in the COPD-OAS group (AHI ≥ 15) had significantly higher BMI, AHI, and a larger neck circumference; however, there were no significant differences in sex, age, and tobacco use between the two groups. Anxiety, depression, hypertension, and diabetes mellitus were more common in participants with overlap syndrome. The quality of life was also poorer in patients with overlapping symptoms, indicated by higher CAT, CCI, and ESS scores. SBQ showed a weak correlation with AHI, and overlap syndrome was more frequent in patients with mild and moderate, compared with severe COPD.

The prevalence of overlap syndrome among patients with COPD in our study was 66%. Steveling et al. reported a prevalence of OSA in patients with COPD of 35% based on an AHI of > 5 events per hour [26], while Hu et al. reported that 68.18% of COPD patients had coexisting OSA (AHI ≥ 5) [27]. In a meta-analysis by Shawon et al., 2.9%–65.9% of patients with COPD had coexisting OSA [7]. The reported prevalence of OSA in patients with COPD thus varies widely, due to different methodologies, diagnostic definitions, and demographic characteristics [27]. However, another survey of inpatients in tertiary hospitals in China found that 77.7% of COPD patients had OSA [28], indicating that overlap syndrome was common among COPD patients in China. These results suggest that family doctors should screen COPD patients for OSA, to improve their quality of life.

The current results showed that BMI and neck circumference were significant predictors for OSA, consistent with other reports [26–29]. This might be because trunk obesity can reduce chest wall compliance and respiratory muscle strength, resulting in ventilation disturbances and ventilation perfusion mismatch [30]. In addition, individuals with larger necks have increased subcutaneous and visceral fat, resulting in crowding of the tonsils, uvula, palatopharyngeal arch, and pharyngeal lateral cord, leaving a narrow gap for airflow and leading to variety of whistling sounds [31, 32]. Peppard and co-workers reported that a 10% increase in weight could lead to a 32% increase in AHI, and a 10% decrease in weight resulted in a 26% decrease in AHI scores [33]. The sex ratio and age were similar in patients with COPD with and without OSA in the current study, possibly because COPD and OSA tend to occur in older men in China [27, 28, 34]. In contrast to other reports however [11, 26], there were no significant differences in mMRC scores, number of acute exacerbations in the year before admission, frequency of hospitalization, proportion of coronary heart disease, and cor pulmonale between COPD patients with and without OSA in the current study. This may be because some information (e.g. mMRC, number of acute exacerbations, etc.) depended on subjective answers by the patients, which could lead to missing reports. A previous study found a missing report rate for acute exacerbation in COPD patients of > 43.0% [35].

We analyzed the risk of OSA in patients with COPD by multiple logistic regression analysis after adjusting for age, sex, BMI, smoking index, and other confounding factors, and showed that hypertension and diabetes were independent risk factors in patients with COPD with coexisting moderate to severe OSA, consistent with other reports [27, 34, 36]. A previous study found that patients with overlap syndrome had more comorbidities, including hypertension and diabetes [30, 34, 37]. Hypertension is the most frequently occurring comorbidity, while diabetes also damages lung function [38].

Multiple regression analysis showed that patients with COPD and coexisting OSA had higher CAT, ESS, CCI, and SBQ scores compared with patients with COPD alone. These findings were in line with previous studies that showed positive relationships between OSA and CAT, ESS, CCI, and SBQ scores in patients with COPD [28, 39]. This suggested that patients with COPD overlapping with OSA had more-severe clinical symptoms, more-limited daily activities, more complications, and poorer sleep quality and quality of life. Although our findings suggested that the SBQ could be used for OSA screening, hypertension and male sex were the main contributory factors, while BMI and neck circumference were less important. This may be related to the generally low BMI of individuals in China [40]. Moreover COPD is a chronic consumptive disease and patients therefore tend to have a low BMI, especially patients with advanced disease [41], which could potentially protect against OSA. But, as a matter of fact, recent studies showed that OSA was more common in COPD patients with low BMI [42]. Soler et al. confirmed that COPD patients with a BMI ≥ 25 kg/m^2^ had a higher risk of OSA [29]. A South Korean clinical study showed that the accuracy of SBQ was not affected after removing the items ‘fatigue’ and ‘neck circumference’ [43], suggesting that the SBQ needs to be further optimized for Asian populations, including modifying or removing the scores for BMI and neck circumference for OSA screening in patients with COPD.

Multiple regression analysis also identified HADS-A and HADS-D scores as independent predictors for OSA in patients with COPD, in accordance with a previous study [37]. Heinzer et al. found that an AHI > 20.6 events per hour of sleep was independently associated with the presence of depression [44]. Numerous studies have examined the interaction between smoking and overlap syndrome, but the results are controversial, with some studies finding a positive relationship [10, 11, 26], while other studies suggested no such relationship [27–29]. Our current results support the latter findings. Although smoking has a pathogenic effect on both COPD and OSA [1, 45], we found no interaction effect on overlap syndrome in patients with COPD in the current study. This may be because smoking resulted in homogenously distributed emphysema, regardless of the severity of smoking [46], and emphysema severity had a protective role for OSA [47].

We found a correlation between COPD severity and OSA. Compared with patients with COPD alone, patients with COPD plus OSA had higher FEV_1_, FEV_1_% of predicted value, and FEV_1_/FVC, and a lower proportion of severe COPD [26–28, 39]. The incidence of OSA increased with increasing airflow restriction in COPD patients with AHI < 5 events per hour, and decreased with increasing airflow restriction in COPD patients with AHI > 30 events per hour. Compared with patients with mild and moderate airflow restriction, the proportion of AHI < 5 events per hour was higher and the proportion of AHI > 30 events per hour was lower in patients with severe airflow restriction. FEV_1_% accounting for the predicted value < 50% was assigned a value of 1 as an independent variable in univariate logistic regression analysis, and the results showed that patients with severe COPD had a lower risk of OSA than those with mild and moderate COPD, after adjusting for age, smoking, CCI, and FEV_1_. A previous study confirmed that hyperinflation had a potential protective effect against OSA in patients with COPD [42, 48]. Lung hyperinflation could prevent the occurrence of OSA by increasing the caudal stretch of the trachea and reducing the critical closing pressure of the upper airway during sleep [13, 49]. Krachman et al. [14]. used quantitative chest computed tomography to evaluate the percentage of emphysema and the degree of gas entrapment in patients with COPD coexisting OSA, and showed that the percentage of emphysema and the degree of gas entrapment were significantly negatively correlated with AHI, indicating the importance of lung volume in maintaining the opening of the upper respiratory tract. Overinflation thus had a protective effect against OSA, and might be a mechanism reducing the risk of OSA in patients with severe COPD.

It was previously shown that COPD patients with overlapping OSA had some independent risk factors; however, the risk of OSA was lower in patients with severe COPD had not been studied. Our study shows that OSA is common in patients with COPD in pulmonary outpatient clinics. In addition, the strengths of this study included the use of a community-based multistage sampling design, large sample size, and random cluster sampling. In addition, all diagnoses of OSA in COPD patients were established by overnight polysomnography which is the gold standard for diagnosing OSA. However, the study also had several limitations. It was a cross-sectional study, and it was therefore not possible to infer any causal relationships. Furthermore, the sample of subjects was not representative of China as a whole. The classifications of OSA, COPD, and overlap syndrome were based on self-reported interview (questionnaire) data, and were therefore subject to recall problems, misunderstanding of the question, and a variety of other factors. In addition, certain medications used to treat COPD, such as theophylline, may also have beneficial effects on OSA [50].

In conclusion, the results of this study showed that COPD patients with overlapping OSA had a poorer quality of life, more daytime sleepiness, and more hypertension than COPD patients without OSA. BMI and CAT and ESS scores were independent risk factors for COPD complicated with OSA. However, the risk of OSA was lower in patients with severe COPD. Our study suggests that OSA is common in patients with COPD in pulmonary outpatient clinics, and pulmonologists should thus consider screening for OSA symptoms in these patients.

## Data Availability

All data relevant to the given manuscript have been stored in a separate file, which can be made freely available to external investigators upon request.
